# Comparison of Modified Manual Acid-Phenol Chloroform Method and Commercial RNA Extraction Kits for Resource Limited Laboratories

**DOI:** 10.1155/2023/9593796

**Published:** 2023-06-09

**Authors:** Samuel Asamoah Sakyi, Alfred Effah, Emmanuel Naturinda, Ebenezer Senu, Stephen Opoku, Benjamin Amoani, Samuel Kekeli Agordzo, Oscar Simon Olympio Mensah, James Grant, Elizabeth Abban, Tonnies Abeku Buckman, Alexander Kwarteng, Richard K. Dadzie Ephraim, Kwabena Owusu Danquah

**Affiliations:** ^1^Department of Molecular Medicine, School of Medicine and Dentistry, Kwame Nkrumah University of Science and Technology, Kumasi, Ghana; ^2^Department of Medical Diagnostics, Faculty of Allied Health Sciences, College of Health Sciences, Kwame Nkrumah University of Science and Technology, Kumasi, Ghana; ^3^Division of Clinical Immunology and Rheumatology, University of Alabama at Birmingham, Birmingham, UK; ^4^Department of Biomedical Science, School of Allied Health Sciences, University of Cape Coast, Cape Coast, Ghana; ^5^Department of Medical Laboratory Technology, Garden City University College, Kumasi, Ghana; ^6^Department of Biochemistry and Biotechnology, Kwame Nkrumah University of Science and Technology, Kumasi, Ghana; ^7^Department of Medical Laboratory Sciences, Faculty of Allied Health, University of Cape Coast, Cape Coast, Ghana; ^8^Department of Clinical Pathology, Noguchi Memorial Institute for Medical Research, University of Ghana, Accra, Ghana

## Abstract

**Method:**

In a comparative experimental cross-sectional study, RNA was extracted from oral swabs and blood samples from 25 healthy individuals at the Department of Molecular Medicine, KNUST. RNA was extracted by the manual AGPC extraction method and commercial RNA extraction kits. The quantity (ng/*μ*l) and purities (260/280 nm) of the extracted RNA were measured spectrophotometrically using the IMPLEN NanoPhotometer® N60. The presence of RNA in the extracts was confirmed using 2% agarose gel electrophoresis. Statistical analyses were conducted using R language.

**Results:**

The yield of RNA extracted from blood and oral swab samples using modified AGPC was significantly higher compared to the commercial methods (*p* < 0.0001). However, the purity of RNA extracted by the manual AGPC method from blood was significantly lower than the commercial methods (*p* < 0.0001). Moreover, the purity from oral swabs using the manual AGPC method was significantly lower compared to QIAamp (*p* < 0.0001) and the OxGEn kits method (*p* < 0.001).

**Conclusion:**

The modified manual AGPC method has a very high yield of RNA extracts using blood samples, which could serve as an alternate cost-effective method for RNA extraction in resource-limited laboratories; however, its purity may not be suitable for downstream processes. Moreover, the manual AGPC method may not be suitable for extracting RNA from oral swab samples. Future investigation is needed to improve the purity of the manual AGPC RNA extraction method and also confirmation of the obtained results by PCR amplification and RNA purity verification by sequencing.

## 1. Introduction

Ribonucleic acid (RNA) is a significant macromolecule that is required for a variety of biological functions, including protein synthesis and catalysis of biological reactions. As a result, extracted RNA is commonly employed in molecular biology tests such as gene expression profiling using reverse-transcription quantitative polymerase chain reaction (RT-qPCR) arrays and next generation sequencing [1]. For efficient RT-PCR assays, quantitative extraction of nucleic acids with high purity from complicated samples is required. Low extraction efficiency could result in distorted signals during exponential amplification, leading to false negative results [2–4]. Low-quality extractions, on the other hand, may contain a variety of PCR inhibitors, resulting in erroneous amplification readouts [4].

RNA extraction is usually performed using one of two methods: phenol-chloroform extraction or commercially available silica spin column extraction. The use of acid guanidinium thiocyanate-phenol-chloroform to induce phase separation of biological mixtures and subsequent selective isolation of molecules of interest is the basis for phenol-chloroform-based RNA extraction [1, 5, 6]. The phenol-chloroform-based RNA extraction method is relatively inexpensive and has higher RNA yield when working with small quantities of cells or tissues [1]. However, the traditional phenol chloroform-based extraction method is time consuming, may necessitate a large volume of blood samples, and involves harmful chemical solvents such as phenol and chloroform [7].

In recent years, commercially available RNA extraction kits have largely replaced conventional RNA extraction approaches. Among them are TRIzol, manufactured by Thermo Fisher Scientific in Waltham, MA, and QIAzol, manufactured by QIAGEN in Hilden, Germany [1], QIAamp® Viral RNA Mini Kit, PureLink RNA Mini Kit (Invitrogen), OxGEn and UltraClean Microbial RNA Isolation Kit (MoBio) [8]. The use of commercial kits necessitates fewer blood samples and takes less time than existing traditional techniques [7]. However, commercial systems and kits are costly and are not readily available in many countries [1, 9]. Many resource-constrained institutions and laboratories lack enough or no research funding, making the use of commercial kits challenging. Even with sufficient funding, using commercial kits can be difficult due to delay in their importation [7]. Phenol-chloroform RNA extraction-based regents can be prepared locally from base chemicals at low cost [5, 6]. Therefore, to promote the use of molecular biology tests such as gene expression profiling using RT-qPCR arrays and next generation sequencing in developing nations [7], where research is less well funded by local governments and agencies, it is critical to improve existing traditional extraction procedures in order to get a sufficient quantity and quality of RNA for downstream processes. In order to develop a proprietary method for RNA extraction from blood and oral swab samples, this study compares a modified standard phenol-chloroform extraction method to commercially available RNA extraction kits; OxGEn and QIAamp® Viral RNA Mini Kit.

## 2. Materials and Methods

### 2.1. Study Design and Setting

This comparative experimental cross-sectional design was carried out on 25 physiologically healthy individuals who consented to participate in the study, from July to October 2022. Blood and oral swab samples were collected for RNA extraction at the Research and Development Unit at the Department of Molecular Medicine, Kwame Nkrumah University of Science and Technology (KNUST). The Department of Molecular Medicine is under the College of Health Sciences at the School of Medicine and Dentistry.

### 2.2. Ethical Consideration

Ethical approval was sought from the Committee on Human Research, Publication and Ethics, School of Medical Sciences, Kwame Nkrumah University of Science and Technology (CHRPE/SMS/KNUST) before the commencement of the study. Study protocol was thoroughly explained to subjects before sample collection. Written informed consent was also sought from participants before sample collection.

### 2.3. Sample Collection

Blood and oral swab samples were collected from participants for RNA extraction. 2 ml of venous blood sample was drawn from each participant into ethylene diamine tetraacetic acid (EDTA) tube and stored at 4°C until assayed. Oral swabs were collected into Eppendorf tubes containing 200 *μ*l of phosphate buffered saline (PBS) and stored at 4°C. RNA was extracted from each sample using three different methods; a modified manual acid-phenol chloroform RNA extraction method, standard protocol for QIAamp® Viral RNA Mini Kit (QIAGEN, Cat. No. 52906) and OxGEn RNA Kit (OxGEn Molecular Solutions, GE-009). Agarose gel electrophoresis was performed to visualize the various RNA extracted.

### 2.4. Manual Acid-Phenol Chloroform Method

#### 2.4.1. Preparation of Home-Made TRIzol Reagent and RBC Lysis Buffer

The reagents involved were prepared in our laboratory under optimum conditions. In the preparation of a 100 ml TRIzol reagent, 38 ml of water saturated phenol (pH 4.3), 5 ml glycerol, and 3.33 ml sodium acetate (pH 5, 3M solution) were measured into a falcon tube. This was followed by the addition of 11.82 g guanidine thiocyanate and 7.61 g ammonium thiocyanate, making a final concentration of 0.8 M and 0.4 M, respectively. Water (ddH_2_O) was added to make 100 ml. The components were mixed by stirring at room temperature until completely dissolved (30–60 mins).

#### 2.4.2. Preparation of 10x Lysis Buffer

89.9 g of NH_4_Cl, 10.0 g KHCO_3,_ and 2.0 ml of 0.5 M EDTA were measured into a flask and dissolved in 800 ml ddH_2_O, and pH was adjusted to 7.3. The volume was brought to 1 liter and mixed thoroughly. This solution is stable for 6 months at 2–8°C in a tightly closed bottle.

#### 2.4.3. Procedure for RNA Extraction by the Manual Acid Phenol Chloroform Method

To 200 *μ*l of sample, 925 *μ*l of the 1X RBC lysis buffer was added and incubated at room temperature for 10 mins. The mixture was centrifuged at 1400 rpm for 10 mins at 25°C, after incubation. The supernatant was discarded, and 1000 *μ*l of 1X RBC lysis buffer was added to the residue. The mixture was allowed to stand for 5 mins at 25°C, followed by centrifugation at 3000 rpm for 2 mins at 25°C. 1000 *μ*l of DPBS was added to the residue and centrifuged at 3000 rpm for 2 mins at 25°C. The supernatant was discarded, and 1200 *μ*l of the home-made TRIzol was added to the residue to resuspend the cells. 200 *μ*l of chloroform (CHCl_3_) was added to the mixture and mixed by vortexing for 15 seconds. The mixture was centrifuged at 13,000 rpm for 10 mins at 4°C. The upper phase was transferred into a new Eppendorf tube, after centrifugation. Equal volume of cold isopropanol was added to the upper phase and inverted to mix. The mixture of the upper phase and isopropanol was placed in a −20°C freezer for 30 mins to enhance precipitation, followed by centrifugation at 13,000 rpm for 10 mins at 4°C. The supernatant was discarded, and 500 *μ*l of ice-cold 75% ethanol was added to the pellet and vortexed and allowed to stand for 10 mins to rinse the pellets (75% ethanol was prepared with RNAse-free water and stored at −20°C). This was followed by centrifugation at 13,000 rpm for 10 mins at 4°C. The supernatant was discarded, and the pellets were air dried for 10 mins. 20 *μ*l of RNAse-free H_2_O was added to the RNA pellet for elution. The RNA was quantified using IMPLEN NanoPhotometer® N60.

### 2.5. Extraction of RNA by QIAamp RNA Mini Kit

The QIAamp kit is designed for the purification of viral RNA from body fluids. In brief, 140 *μ*l of the sample (blood or oral swab) was transferred to 560 *μ*l buffer AVL-carrier RNA in a microcentrifuge tube. This was followed by addition of 560 *μ*l of ethanol (96–100%) after 10 mins of incubation. 630 *μ*l of the solution was transferred to the QIAamp Mini column followed by centrifugation at 6000 ×g (8000 rpm) for 1 minute. The QIAamp Mini column was placed into a clean 2 ml collection tube. This step was repeated until all of the lysate had been loaded onto the spin column. 500 *μ*l of wash buffer AW1 was added to the QIAamp Mini column and centrifuged at 6000 ×g (8000 rpm) for 1 minute. The collection tube was discarded and replaced with a clean 2 ml collection tube. 500 *μ*l of wash buffer AW2 was added to the QIAamp mini column and centrifuged at full speed (20,000 × g; 14,000 rpm) for 3 minutes. 60 *μ*l of buffer AVE was used for the elution of the RNA.

### 2.6. Extraction of RNA by OxGEn Kit

The OxGEn kit is a spin column-based RNA extraction kit. 560 *μ*l Solution A was transferred into a 1.5 ml microcentrifuge tube, 5 *μ*l of RNA carrier was added, 140 *μ*l of the sample was added to the tube and mixed by pulse-vortex for 15 s, followed by incubation at room temperature. 560 *μ*l of ethanol (96–100%) was added to the mixture. 650 *μ*l of the lysate was transferred onto the G-spin column and centrifuged at 8000 rpm for 1 minute. This step was repeated with the remaining lysate until the entire lysate had passed through the G-spin column. The column was washed with 600 *μ*l of solution W1 and centrifuged at 8000 rpm for 1 min. The column was washed with 600 *μ*l of solution W2 followed by centrifugation at 13000 rpm for 1 minute, and the collection tube was replaced with a new one. The residual wash buffer was removed by centrifuging at 13000 rpm for 2 minutes, and the column was transferred onto a new 1.5 ml microcentrifuge tube. 50 *μ*l of solution E was added onto the column, followed by incubation for 3 min at room temperature, RNA was eluted by spinning down at 8000 rpm for 1 minute, and RNA was quantified using the IMPLEN Nanophotometer.

### 2.7. Analysis of RNA Extract

Both the concentration and absorbance ratio at A260/280 nm were measured using the IMPLEN NanoPhotometer® N60. The concentrations were estimated in ng/*μ*l, followed by analysis on a 2% agarose gel electrophoresis.

#### 2.7.1. Statistical Analyses

Data from the study was entered into Microsoft Excel 2019. Statistical analyses were performed on R language for statistical computing [10]. Distribution and levels of RNA concentration and purity between blood and oral swabs were present by the kernel density plot and boxplot and the subsequent Mann Whitney U test. Comparison of RNA concentration and purity between the manual AGPC method and the two commercial extraction kits from blood and oral swabs were represented by boxplot; Kruskal–Wallis tests and subsequent post-hoc tests were used for the statistical comparisons. *p* value of <0.05 was considered statistically significant.

## 3. Results

### 3.1. Comparison of Both RNA Quality and Yield between Samples

**Figure 1:**
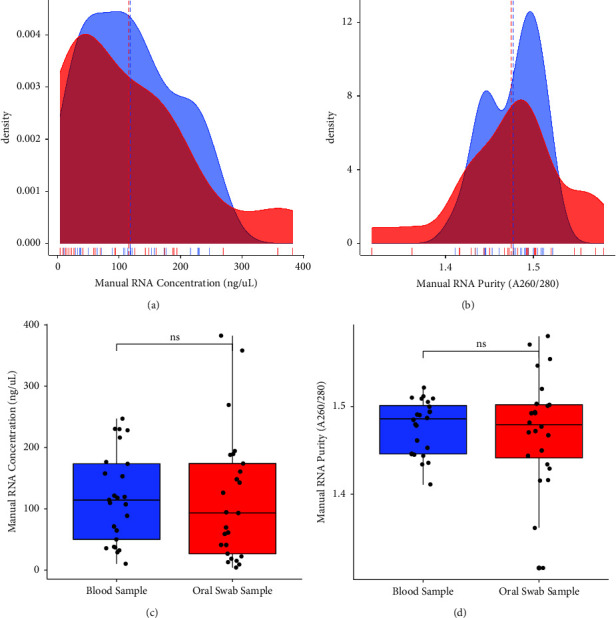
Comparison of RNA concentration (a and c) and purity (b and d) between blood and oral swab samples for the manual AGPC RNA extraction method; ns: not significant.

Figure 1 shows both concentrations (A and C) and purity (B and D) of RNA using the manual AGPC RNA extraction method. Of comparison of RNA yield concentration between blood samples and oral swabs, blood had slightly higher yield compared to that of oral swab samples. However, there was no significant difference between the concentrations of RNA in these samples (*p* = 0.525) (Figure 1(c)**)**. Moreover, the purity of RNA was slightly higher in blood compared to oral swabs, although the purity of RNA between these two samples was not statistically significant (*p* = 0.740) (Figure 1(d)).

### 3.2. Comparison of Yield and Purity between Methods Using Blood Samples

**Figure 2:**
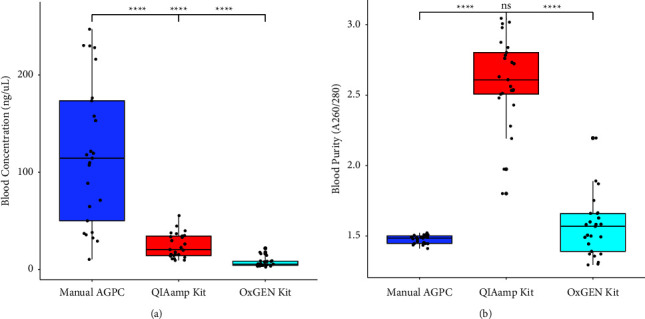
Box and Whisker plot of RNA concentration (a) and purity (b) based on the three different methods, using blood samples; ^*∗∗∗∗*^*p* < 0.0001, ^*∗∗∗*^*p* < 0.001, ^*∗∗*^*p* < 0.01, and ^*∗*^*p* < 0.05; ns: not significant.

In comparison of yields, all the three methods produced significantly different yields of RNA extracts from blood samples (*p* < 0.0001). In a post-hoc test, the yield using modified manual acid phenol chloroform was significantly higher compared to the two commercial methods (QIAamp method and OxGEn kit) (*p* < 0.0001). Moreover, the yield between the QIAamp method and the OxGEn kit was also significantly different (*p* < 0.0001) (Figure 2(a)).

In comparison of purity, the purity of RNA extracted by the manual AGPC method was significantly lower than that extracted by the QIAamp method (*p* < 0.0001). However, the purity between the manual AGPC and OxGEn kit was similar (*p* > 0.05). The QIAamp kit also produced significantly higher purity of RNA extracts from that of the OxGEn kit method (*p* < 0.0001) (Figure 2(b)).

### 3.3. Comparison of Yield and Purity between Methods Using Oral Swabs

**Figure 3:**
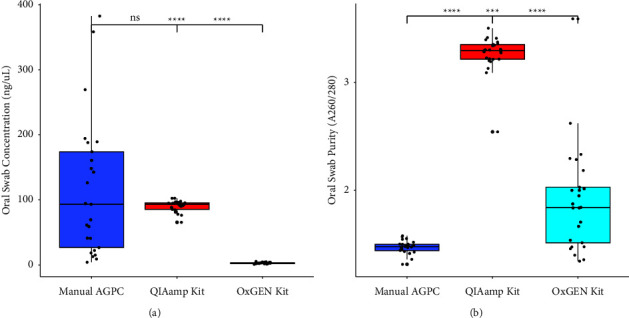
Box and Whisker plot of RNA concentration (a) and purity (b) based on the three different methods, using oral swabs; ^*∗∗∗∗*^*p* < 0.0001, ^*∗∗∗*^*p* < 0.001, ^*∗∗*^*p* < 0.01, and ^*∗*^*p* < 0.05; ns: not significant.

The yield using the manual AGPC method was significantly higher than that of the OxGEn kit method (*p* < 0.0001) using the oral swabs. In addition, the yield produced using the QIAamp kit was significantly higher compared to the concentration of RNA extracted using the OxGEn kit method using the oral swabs. However, the yield using either the manual AGPC method or the QIAamp kit method was proportional (*p* > 0.05) (Figure 3(a)).

In comparison of purity of RNA extracted, the purity of RNA extracted using the manual AGPC method was significantly lower compared to using the QIAamp kit (*p* < 0.0001) and the OxGEn kit method (*p* < 0.001). However, the RNA extracted using the QIAamp kit method was significantly pure than using the OxGEn kit method (*p* < 0.0001) (Figure 3(b)).

### 3.4. Agarose Gel Electrophoresis of RNA Extracts

**Figure 4:**
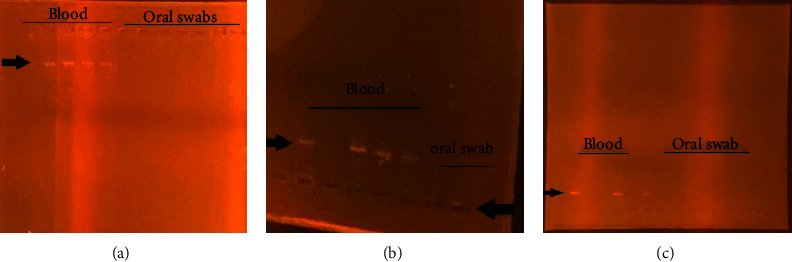
Gel electrophoresis of RNAs isolated by manual AGPC (a), QIAamp kit (b), and OxGEN kit (c).

Following agarose gel electrophoresis, there were strong bands for RNA extracted from blood samples by each of the three methods; manual AGPC method, QIAamp kit, and OxGEn (Figure 4). Visibility of the RNA bands on the gel was comparable between the manual method and the commercial methods (QIAamp kit and OxGEn kit), despite differences in blood RNA concentration produced by these methods. However, there were no visible bands of RNA extracted by the manual AGPC method and the OxGEn kit using oral swab samples (Figures 4(a) and 4(c)), while the QIAamp kit produced faint bands (Figure 4(b)).

### 3.5. Comparison of Cost Involved in Using the Three Different Methods

Table 1 shows the cost involved in extracting 50 samples using each method. At the time of purchasing the various items, a dollar was equivalent to GH₵ 9.9 Ghana Cedis. It required $1.85 to prepare 60 ml of homemade TRIzol and $3.03 for 500 ml of the RBC Lysis buffer required for the extraction of 50 sample (25 blood and 25 oral swabs) using the manual AGPC method. However, an amount of $314 [11] and $113.04 was required for the extraction of the same number of samples by the QIAamp method and the OxGEn kit, respectively (Table 1).

## 4. Discussion

RNA extraction is usually conducted using one of two methods: phenol-chloroform extraction or commercially available silica spin column extraction. In recent years, commercially available RNA extraction kits have largely replaced conventional RNA extraction approaches [7]. However, commercial systems and kits are costly, and they are not widely available in many countries [1, 9]. Many resource-constrained institutions and laboratories lack enough or no research funding, making the use of commercial kits challenging. Cost-effectiveness, availability, dependability, and purity are all needs for small laboratories. Even with sufficient funding, using commercial kits can be difficult owing to time constraints and technological limitations [7]. Moreover, phenol-chloroform RNA extraction-based reagents can be prepared locally from base chemicals at low cost [5, 6]. It is against this background that this study compared the quality and quantity of RNA recovered from the modified manual acid guanidinium thiocyanate-phenol-chloroform (AGPC) extraction method and commercial RNA extraction kits (QIAamp Viral RNA mini kit and OxGEn kit) to develop a proprietary method for RNA extraction from both blood and oral swabs. All the reagents involved in this manual extraction method were locally prepared from their basic chemical constituents.

In this study, RNA recovered from twenty-five blood samples using the manual AGPC method had the highest concentration of RNA with a median value of 114.40 ng/*μ*l compared to that of QIAamp (20.4 ng/*µ*l) and OxGEn kit (5.32 ng/*μ*l). However, the purity (A260/280 ratio) of RNA extracted from blood by QIAamp and OxGEn was purer (≥1.8) compared to that of the modified manual AGPC method (≤1.8). Although the QIAamp and OxGEn kit produced high purity of RNA extracts, both kits do not meet the demands of cost effectiveness, costing $314 and $113.04, respectively, to extract 50 samples each [11], whereas at an equivalent price, the manual extraction method can extract over 3000 samples. Surprisingly, the 260/230 ratios determined by all the three methods were significantly lower than the optimum. These low values are usually attributed to salt contamination, despite the fact that the final washing step in both extraction methods requires over 70% ethanol wash. Ionic strength, on the other hand, is known to influence nucleic acid absorbance, particularly at 260 nm, which could have influenced the 260/230 ratio [12, 13]. For oral swab samples, both the manual and the QIAamp methods yielded comparable RNA concentrations, with the QIAamp mini kit yielding higher purity than both the manual AGPC method and the OxGEn kit. In our attempt to address the issue with purity, various modifications were made including repeated 75% ethanol wash. However, this decreased the concentration of the extract without any concurrent increase in the purity, contrasting what was stated in a study conducted by Toni et al. [1]. The manual AGPC method's high yield may be attributed to the larger volume of blood used than that used for the commercial kits, 200 *μ*l and 140 *μ*l, respectively. Interestingly, similar volumes of oral swabs were used, but comparable concentrations were produced by both the manual AGPC and QIAamp kits. As a result, the difference in RNA concentration recovered by each method cannot be explained solely by the volumes used. However, the high concentration observed with the modified manual AGPC method can be explained by the fact that it extracts total RNA from the samples, whereas the QIAamp and OxGEn kits are designed specifically for viral RNA extraction. Extracted RNA was verified by 2% agarose gel electrophoresis. For blood samples, RNA recovered by either the manual AGPC or QIAamp method produced comparable visible RNA bands on the gel, despite the differences in concentrations. By contrast, none of the RNA from oral swab RNA extracts by the manual AGPC method and OxGEn produced bands on the gel. RNA extracts by the QIAamp method had faint bands on the gel.

In agreement with the findings of this study, the manual AGPC RNA extraction method has been demonstrated to yield high quantity of RNA compared to column-based kits including the QIAamp and OxGEn kit using different samples [6, 14, 15]. Several studies have also shown that manual AGPC-based RNA extraction yields 2.4–93 times more RNA than silica column-based protocols [16–18], which is consistent with our findings. Additional advantage is that since only the aqueous phase which contains principally RNA after addition of chloroform is used to obtain the final RNA, there is low or insignificant levels of DNA contamination [19]. On the contrary, few studies have produced conflicting results; for example, Xiang et al. found no significant difference in RNA purity between RNeasy, a silica column-based as QIAamp Viral RNA mini kit and OxGEn, and manual AGPC using sputum samples, although the later yielded high concentration of RNA [18]. Although the manual AGPC method could yield a high quantity of RNA, this method is time consuming and involves numerous steps, which often result in contamination of the RNA. The use of AGPC to induce phase separation of biological mixtures and subsequent selective isolation of molecules of interest requires toxic reagents such as phenol and chloroform [1, 6]. Both phenol and chloroform, aside posing danger to human health, can remain significant contaminants in RNA extraction. The presence of these contaminants may have an impact on both the RNA quantification on spectrophotometers and the results of subsequent experiments.

Furthermore, the lack of significance in comparing yield and purity of RNA extracts in the aforementioned studies may validate the idea that traditional methods, when modified and carefully monitored, will be of great use in RNA extraction from blood and oral swabs, particularly in resource-constrained settings where there is a need to reduce cost but with high efficiency. One major limitation of this study is the lack of confirmation of the RNA extracts by PCR or sequencing. The study could not confirm the RNA obtained by the manual AGPC method by either PCR or sequencing due to limited financial resources.

## 5. Conclusion

The modified manual AGPC method has a very high yield of RNA extracts using blood samples, which could serve as an alternate cost-effective method for RNA extraction in resource-limited laboratories; however, its purity may not be suitable for downstream processes. Moreover, the manual AGPC method may not be suitable for extracting RNA from oral swab samples. Future investigation is needed to improve the purity of the manual AGPC RNA extraction method and also confirmation of the obtained results by PCR amplification and RNA purity verification by sequencing.

## Figures and Tables

**Table 1:** This shows the cost involved in extracting 50 samples using each method.

Item	Quantity	Price
Homemade TRIzol	60 ml	$1.85 (GH₵18.4)
Lysis buffer	500 ml	$3.03 (GH₵30.45)
**Total**		**$4.88 (GH₵48.85)**
QIAamp viral RNA kit	1 box	**$314 (GH₵3158.81)**
OxGEn kit	1 box	**$113.04 (GH₵1119.1)**

*Note*. Bold values represent significant difference at *p* < 0.0001.

## Data Availability

All data generated or analyzed during this study are included within this article. The data can be obtained from the corresponding author upon reasonable request.
